# Supplementary material for: Modeling glioblastoma heterogeneity as a dynamic network of cell states
Source: Mol Syst Biol. 2021 Sep 16;17(9):e10105. doi: 10.15252/msb.202010105 (PMC8444284; doi:10.15252/msb.202010105)
Supplement: Supplementary file 5 — Source Data for Figure 3 [file MSB-17-e10105-s001.zip › Figure3A_sourcedata/GSEA_3065/nosigdb_state5.GseaPreranked.1622718435914/RICHARDS_NOWAKOWSKI_SCIENCE_2017_MGE_RG2_UPREG.html]

Details for gene set RICHARDS\_NOWAKOWSKI\_SCIENCE\_2017\_MGE\_RG2\_UPREG[GSEA]

|  || Dataset | state5 |
| Phenotype | NoPhenotypeAvailable |
| Upregulated in class | na\_neg |
| GeneSet | RICHARDS\_NOWAKOWSKI\_SCIENCE\_2017\_MGE\_RG2\_UPREG |
| Enrichment Score (ES) | -0.56613535 |
| Normalized Enrichment Score (NES) | -2.2163801 |
| Nominal p-value | 0.0 |
| FDR q-value | 0.0 |
| FWER p-Value | 0.0 |
Table: GSEA Results Summary

  

Fig 1: Enrichment plot: RICHARDS\_NOWAKOWSKI\_SCIENCE\_2017\_MGE\_RG2\_UPREG      
 Profile of the Running ES Score & Positions of GeneSet Members on the Rank Ordered List

  

| PROBE | GENE SYMBOL | GENE\_TITLE | RANK IN GENE LIST | RANK METRIC SCORE | RUNNING ES | CORE ENRICHMENT || 1 | BCAN |  |  | 15 | 0.850 | 0.0049 | No |
| 2 | CD63 |  |  | 24 | 0.769 | 0.0100 | No |
| 3 | PDIA6 |  |  | 29 | 0.742 | 0.0153 | No |
| 4 | HSP90B1 |  |  | 33 | 0.729 | 0.0207 | No |
| 5 | HSPA5 |  |  | 43 | 0.698 | 0.0251 | No |
| 6 | TNC |  |  | 45 | 0.680 | 0.0303 | No |
| 7 | PDIA3 |  |  | 49 | 0.652 | 0.0350 | No |
| 8 | PRDX4 |  |  | 80 | 0.553 | 0.0359 | No |
| 9 | PON2 |  |  | 84 | 0.546 | 0.0398 | No |
| 10 | SEMA5A |  |  | 111 | 0.516 | 0.0408 | No |
| 11 | ATP1B3 |  |  | 113 | 0.510 | 0.0447 | No |
| 12 | BSG |  |  | 135 | 0.493 | 0.0461 | No |
| 13 | CCDC14 |  |  | 136 | 0.492 | 0.0499 | No |
| 14 | FAT1 |  |  | 155 | 0.478 | 0.0515 | No |
| 15 | SLC16A1 |  |  | 186 | 0.449 | 0.0516 | No |
| 16 | PNN |  |  | 195 | 0.442 | 0.0541 | No |
| 17 | RCN1 |  |  | 217 | 0.427 | 0.0550 | No |
| 18 | PABPN1 |  |  | 237 | 0.414 | 0.0560 | No |
| 19 | EMP2 |  |  | 239 | 0.414 | 0.0591 | No |
| 20 | TMX1 |  |  | 255 | 0.406 | 0.0605 | No |
| 21 | HELLS |  |  | 280 | 0.393 | 0.0608 | No |
| 22 | SCD |  |  | 284 | 0.391 | 0.0635 | No |
| 23 | RIC3 |  |  | 308 | 0.378 | 0.0638 | No |
| 24 | ALG8 |  |  | 310 | 0.377 | 0.0666 | No |
| 25 | CHD7 |  |  | 312 | 0.376 | 0.0694 | No |
| 26 | CREB5 |  |  | 321 | 0.373 | 0.0714 | No |
| 27 | FKBP10 |  |  | 337 | 0.365 | 0.0725 | No |
| 28 | NOTCH1 |  |  | 348 | 0.361 | 0.0742 | No |
| 29 | LDLR |  |  | 404 | 0.339 | 0.0704 | No |
| 30 | AASS |  |  | 494 | 0.313 | 0.0624 | No |
| 31 | CDCA7 |  |  | 495 | 0.313 | 0.0649 | No |
| 32 | MSI2 |  |  | 496 | 0.313 | 0.0673 | No |
| 33 | BRCA1 |  |  | 522 | 0.305 | 0.0668 | No |
| 34 | AHI1 |  |  | 609 | 0.283 | 0.0589 | No |
| 35 | KNTC1 |  |  | 622 | 0.281 | 0.0597 | No |
| 36 | HES6 |  |  | 653 | 0.274 | 0.0584 | No |
| 37 | JAM2 |  |  | 675 | 0.270 | 0.0580 | No |
| 38 | ZKSCAN1 |  |  | 678 | 0.269 | 0.0599 | No |
| 39 | SPECC1 |  |  | 689 | 0.266 | 0.0608 | No |
| 40 | ZBTB20 |  |  | 696 | 0.263 | 0.0621 | No |
| 41 | PDPN |  |  | 719 | 0.258 | 0.0616 | No |
| 42 | TMEM18 |  |  | 764 | 0.248 | 0.0584 | No |
| 43 | ORC6 |  |  | 806 | 0.240 | 0.0555 | No |
| 44 | PAM |  |  | 844 | 0.233 | 0.0530 | No |
| 45 | ZNF121 |  |  | 868 | 0.230 | 0.0521 | No |
| 46 | SCAF11 |  |  | 872 | 0.229 | 0.0535 | No |
| 47 | WEE1 |  |  | 919 | 0.222 | 0.0499 | No |
| 48 | C9orf3 |  |  | 934 | 0.220 | 0.0500 | No |
| 49 | ILDR2 |  |  | 965 | 0.215 | 0.0481 | No |
| 50 | TMEM97 |  |  | 995 | 0.211 | 0.0464 | No |
| 51 | PRKDC |  |  | 1051 | 0.204 | 0.0416 | No |
| 52 | SLC35F1 |  |  | 1092 | 0.199 | 0.0384 | No |
| 53 | MMS22L |  |  | 1126 | 0.195 | 0.0361 | No |
| 54 | GLCCI1 |  |  | 1172 | 0.188 | 0.0323 | No |
| 55 | NASP |  |  | 1225 | 0.182 | 0.0276 | No |
| 56 | SRSF10 |  |  | 1270 | 0.176 | 0.0239 | No |
| 57 | TMEM123 |  |  | 1293 | 0.172 | 0.0227 | No |
| 58 | SOAT1 |  |  | 1400 | 0.162 | 0.0115 | No |
| 59 | CDC37 |  |  | 1410 | 0.161 | 0.0117 | No |
| 60 | FOS |  |  | 1412 | 0.161 | 0.0129 | No |
| 61 | CEP152 |  |  | 1436 | 0.159 | 0.0114 | No |
| 62 | AXL |  |  | 1495 | 0.153 | 0.0058 | No |
| 63 | CENPF |  |  | 1531 | 0.149 | 0.0029 | No |
| 64 | EGFR |  |  | 1547 | 0.147 | 0.0023 | No |
| 65 | SRSF6 |  |  | 1558 | 0.146 | 0.0023 | No |
| 66 | RBL1 |  |  | 1669 | 0.136 | -0.0095 | No |
| 67 | FAM161A |  |  | 1697 | 0.133 | -0.0117 | No |
| 68 | IGSF11 |  |  | 1763 | 0.129 | -0.0182 | No |
| 69 | BRIP1 |  |  | 1764 | 0.129 | -0.0172 | No |
| 70 | MIS18BP1 |  |  | 1765 | 0.129 | -0.0162 | No |
| 71 | BRCA2 |  |  | 1781 | 0.128 | -0.0170 | No |
| 72 | CYR61 |  |  | 1850 | 0.123 | -0.0240 | No |
| 73 | HNRNPA2B1 |  |  | 1852 | 0.122 | -0.0231 | No |
| 74 | RFTN2 |  |  | 1867 | 0.121 | -0.0238 | No |
| 75 | PCDH17 |  |  | 1889 | 0.120 | -0.0253 | No |
| 76 | WRN |  |  | 1933 | 0.117 | -0.0295 | No |
| 77 | SRRM2 |  |  | 1935 | 0.117 | -0.0287 | No |
| 78 | CD99 |  |  | 1945 | 0.116 | -0.0288 | No |
| 79 | NOTCH3 |  |  | 1978 | 0.113 | -0.0317 | No |
| 80 | LIMD1 |  |  | 2036 | 0.109 | -0.0375 | No |
| 81 | TSPAN6 |  |  | 2087 | 0.106 | -0.0425 | No |
| 82 | TRMT10B |  |  | 2108 | 0.105 | -0.0440 | No |
| 83 | FUT8 |  |  | 2157 | 0.101 | -0.0489 | No |
| 84 | ING5 |  |  | 2192 | 0.099 | -0.0521 | No |
| 85 | C1orf35 |  |  | 2269 | 0.094 | -0.0602 | No |
| 86 | DYNC2H1 |  |  | 2278 | 0.093 | -0.0604 | No |
| 87 | SRSF1 |  |  | 2334 | 0.090 | -0.0662 | No |
| 88 | EGR1 |  |  | 2336 | 0.090 | -0.0656 | No |
| 89 | NUP43 |  |  | 2383 | 0.087 | -0.0703 | No |
| 90 | NOP56 |  |  | 2390 | 0.086 | -0.0703 | No |
| 91 | CENPC |  |  | 2392 | 0.086 | -0.0697 | No |
| 92 | NES |  |  | 2417 | 0.084 | -0.0719 | No |
| 93 | MDK |  |  | 2458 | 0.082 | -0.0759 | No |
| 94 | HAT1 |  |  | 2459 | 0.082 | -0.0753 | No |
| 95 | POLI |  |  | 2471 | 0.081 | -0.0759 | No |
| 96 | PHGDH |  |  | 2485 | 0.081 | -0.0768 | No |
| 97 | MTCL1 |  |  | 2497 | 0.079 | -0.0775 | No |
| 98 | ATAD2 |  |  | 2545 | 0.077 | -0.0824 | No |
| 99 | TMA16 |  |  | 2578 | 0.075 | -0.0855 | No |
| 100 | PIKFYVE |  |  | 2596 | 0.074 | -0.0870 | No |
| 101 | CCDC138 |  |  | 2757 | 0.065 | -0.1052 | No |
| 102 | TRIM16 |  |  | 2768 | 0.064 | -0.1058 | No |
| 103 | PSD3 |  |  | 2778 | 0.064 | -0.1064 | No |
| 104 | MNS1 |  |  | 2792 | 0.063 | -0.1074 | No |
| 105 | FAM111A |  |  | 2820 | 0.062 | -0.1101 | No |
| 106 | RAD18 |  |  | 2825 | 0.061 | -0.1101 | No |
| 107 | FDPS |  |  | 2849 | 0.060 | -0.1123 | No |
| 108 | CHEK1 |  |  | 2877 | 0.059 | -0.1150 | No |
| 109 | RAD51AP1 |  |  | 2905 | 0.057 | -0.1177 | No |
| 110 | DCAF16 |  |  | 2937 | 0.056 | -0.1209 | No |
| 111 | HAUS5 |  |  | 2983 | 0.053 | -0.1257 | No |
| 112 | E2F7 |  |  | 2989 | 0.052 | -0.1259 | No |
| 113 | SFRP2 |  |  | 2997 | 0.052 | -0.1263 | No |
| 114 | TCF7L1 |  |  | 3012 | 0.051 | -0.1276 | No |
| 115 | EPHB2 |  |  | 3015 | 0.051 | -0.1274 | No |
| 116 | ABAT |  |  | 3020 | 0.051 | -0.1275 | No |
| 117 | HDAC9 |  |  | 3051 | 0.049 | -0.1306 | No |
| 118 | NSRP1 |  |  | 3092 | 0.047 | -0.1349 | No |
| 119 | SRSF7 |  |  | 3126 | 0.046 | -0.1384 | No |
| 120 | FANCD2 |  |  | 3151 | 0.044 | -0.1409 | No |
| 121 | LIG1 |  |  | 3181 | 0.042 | -0.1439 | No |
| 122 | RFC4 |  |  | 3212 | 0.041 | -0.1471 | No |
| 123 | CORO1C |  |  | 3217 | 0.040 | -0.1473 | No |
| 124 | UTP20 |  |  | 3228 | 0.040 | -0.1481 | No |
| 125 | CNTLN |  |  | 3231 | 0.040 | -0.1481 | No |
| 126 | LARP1B |  |  | 3242 | 0.039 | -0.1489 | No |
| 127 | TFDP2 |  |  | 3271 | 0.038 | -0.1519 | No |
| 128 | CTPS1 |  |  | 3314 | 0.036 | -0.1565 | No |
| 129 | MTA3 |  |  | 3325 | 0.035 | -0.1574 | No |
| 130 | GAB1 |  |  | 3372 | 0.033 | -0.1626 | No |
| 131 | C19orf48 |  |  | 3373 | 0.033 | -0.1623 | No |
| 132 | SMC2 |  |  | 3374 | 0.032 | -0.1621 | No |
| 133 | FANCI |  |  | 3375 | 0.032 | -0.1618 | No |
| 134 | FRMD5 |  |  | 3383 | 0.032 | -0.1624 | No |
| 135 | CTNNB1 |  |  | 3417 | 0.031 | -0.1660 | No |
| 136 | G2E3 |  |  | 3493 | 0.028 | -0.1745 | No |
| 137 | ESCO2 |  |  | 3672 | 0.021 | -0.1952 | No |
| 138 | SMC1A |  |  | 3696 | 0.020 | -0.1977 | No |
| 139 | PRIM1 |  |  | 3702 | 0.020 | -0.1982 | No |
| 140 | MSH6 |  |  | 3736 | 0.019 | -0.2019 | No |
| 141 | BLM |  |  | 3771 | 0.017 | -0.2057 | No |
| 142 | ITGB3BP |  |  | 3830 | 0.014 | -0.2124 | No |
| 143 | NUP62 |  |  | 3839 | 0.014 | -0.2132 | No |
| 144 | BAG4 |  |  | 3843 | 0.014 | -0.2135 | No |
| 145 | CDC7 |  |  | 3855 | 0.014 | -0.2146 | No |
| 146 | RFC3 |  |  | 3863 | 0.013 | -0.2154 | No |
| 147 | DIS3L |  |  | 3881 | 0.013 | -0.2173 | No |
| 148 | CEP83 |  |  | 3941 | 0.010 | -0.2241 | No |
| 149 | KIF15 |  |  | 3950 | 0.009 | -0.2249 | No |
| 150 | NEDD1 |  |  | 3958 | 0.009 | -0.2257 | No |
| 151 | LIN54 |  |  | 3974 | 0.008 | -0.2274 | No |
| 152 | MIS12 |  |  | 3994 | 0.007 | -0.2296 | No |
| 153 | NCAPD3 |  |  | 3996 | 0.007 | -0.2296 | No |
| 154 | TLE3 |  |  | 4004 | 0.007 | -0.2304 | No |
| 155 | PBX3 |  |  | 4054 | 0.005 | -0.2361 | No |
| 156 | CCNE2 |  |  | 4180 | -0.001 | -0.2507 | No |
| 157 | LIPG |  |  | 4186 | -0.001 | -0.2513 | No |
| 158 | WDHD1 |  |  | 4234 | -0.003 | -0.2567 | No |
| 159 | PRC1 |  |  | 4241 | -0.003 | -0.2574 | No |
| 160 | PARD3 |  |  | 4265 | -0.004 | -0.2601 | No |
| 161 | SOX2 |  |  | 4271 | -0.005 | -0.2606 | No |
| 162 | ZC3HAV1 |  |  | 4394 | -0.010 | -0.2748 | No |
| 163 | CDK2 |  |  | 4489 | -0.013 | -0.2857 | No |
| 164 | MCM4 |  |  | 4534 | -0.015 | -0.2907 | No |
| 165 | CCND2 |  |  | 4539 | -0.016 | -0.2911 | No |
| 166 | MCM10 |  |  | 4542 | -0.016 | -0.2912 | No |
| 167 | C5orf30 |  |  | 4578 | -0.017 | -0.2952 | No |
| 168 | FOXN3 |  |  | 4639 | -0.020 | -0.3020 | No |
| 169 | CENPK |  |  | 4675 | -0.021 | -0.3059 | No |
| 170 | WDR76 |  |  | 4723 | -0.023 | -0.3113 | No |
| 171 | TRIM26 |  |  | 4730 | -0.023 | -0.3118 | No |
| 172 | CDK1 |  |  | 4766 | -0.025 | -0.3157 | No |
| 173 | HAUS6 |  |  | 4771 | -0.025 | -0.3160 | No |
| 174 | BARD1 |  |  | 4776 | -0.025 | -0.3162 | No |
| 175 | CDO1 |  |  | 4786 | -0.025 | -0.3171 | No |
| 176 | FAM111B |  |  | 4792 | -0.025 | -0.3175 | No |
| 177 | MCM2 |  |  | 4824 | -0.027 | -0.3209 | No |
| 178 | POLA1 |  |  | 4853 | -0.028 | -0.3240 | No |
| 179 | SF3A3 |  |  | 4875 | -0.028 | -0.3262 | No |
| 180 | DTL |  |  | 4909 | -0.029 | -0.3298 | No |
| 181 | NUP153 |  |  | 4929 | -0.030 | -0.3318 | No |
| 182 | MCM7 |  |  | 4986 | -0.032 | -0.3381 | No |
| 183 | TRIM9 |  |  | 5014 | -0.034 | -0.3410 | No |
| 184 | SKP2 |  |  | 5018 | -0.034 | -0.3411 | No |
| 185 | PPIL4 |  |  | 5022 | -0.034 | -0.3412 | No |
| 186 | GINS2 |  |  | 5024 | -0.034 | -0.3410 | No |
| 187 | PTPN13 |  |  | 5065 | -0.035 | -0.3454 | No |
| 188 | PRMT5 |  |  | 5155 | -0.039 | -0.3555 | No |
| 189 | APBB2 |  |  | 5184 | -0.039 | -0.3585 | No |
| 190 | TP53 |  |  | 5275 | -0.043 | -0.3687 | No |
| 191 | STK39 |  |  | 5333 | -0.046 | -0.3750 | No |
| 192 | UHRF1 |  |  | 5337 | -0.046 | -0.3750 | No |
| 193 | NUSAP1 |  |  | 5389 | -0.048 | -0.3806 | No |
| 194 | POLD3 |  |  | 5393 | -0.048 | -0.3806 | No |
| 195 | C12orf29 |  |  | 5541 | -0.055 | -0.3973 | No |
| 196 | YAP1 |  |  | 5575 | -0.056 | -0.4007 | No |
| 197 | STAG2 |  |  | 5601 | -0.057 | -0.4032 | No |
| 198 | CLSPN |  |  | 5614 | -0.058 | -0.4042 | No |
| 199 | CHIC2 |  |  | 5655 | -0.060 | -0.4084 | No |
| 200 | TPM2 |  |  | 5660 | -0.060 | -0.4084 | No |
| 201 | ALDH6A1 |  |  | 5682 | -0.061 | -0.4104 | No |
| 202 | QKI |  |  | 5715 | -0.062 | -0.4136 | No |
| 203 | ARHGAP5 |  |  | 5716 | -0.063 | -0.4131 | No |
| 204 | TIMELESS |  |  | 5717 | -0.063 | -0.4126 | No |
| 205 | CRKL |  |  | 5741 | -0.063 | -0.4148 | No |
| 206 | KIF22 |  |  | 5758 | -0.064 | -0.4162 | No |
| 207 | PDLIM3 |  |  | 5938 | -0.071 | -0.4366 | No |
| 208 | SEPT10 |  |  | 5944 | -0.072 | -0.4366 | No |
| 209 | PRTFDC1 |  |  | 5982 | -0.073 | -0.4404 | No |
| 210 | BAZ1A |  |  | 6008 | -0.074 | -0.4427 | No |
| 211 | RFC1 |  |  | 6011 | -0.074 | -0.4423 | No |
| 212 | EXOSC9 |  |  | 6019 | -0.075 | -0.4426 | No |
| 213 | EXOSC8 |  |  | 6020 | -0.075 | -0.4420 | No |
| 214 | DKC1 |  |  | 6027 | -0.076 | -0.4421 | No |
| 215 | SFRP1 |  |  | 6042 | -0.076 | -0.4431 | No |
| 216 | BMS1 |  |  | 6105 | -0.080 | -0.4498 | No |
| 217 | PDLIM5 |  |  | 6127 | -0.081 | -0.4516 | No |
| 218 | LRR1 |  |  | 6149 | -0.082 | -0.4534 | No |
| 219 | G3BP1 |  |  | 6152 | -0.082 | -0.4530 | No |
| 220 | CHAF1A |  |  | 6157 | -0.082 | -0.4528 | No |
| 221 | RFC5 |  |  | 6186 | -0.084 | -0.4554 | No |
| 222 | PAICS |  |  | 6198 | -0.085 | -0.4561 | No |
| 223 | MKI67 |  |  | 6211 | -0.086 | -0.4568 | No |
| 224 | TROVE2 |  |  | 6233 | -0.087 | -0.4586 | No |
| 225 | SNX4 |  |  | 6247 | -0.087 | -0.4594 | No |
| 226 | MRPL45 |  |  | 6248 | -0.087 | -0.4587 | No |
| 227 | RRM1 |  |  | 6298 | -0.090 | -0.4637 | No |
| 228 | SMAD5 |  |  | 6355 | -0.093 | -0.4696 | No |
| 229 | TLK1 |  |  | 6386 | -0.094 | -0.4723 | No |
| 230 | DAXX |  |  | 6440 | -0.098 | -0.4778 | No |
| 231 | IQGAP2 |  |  | 6450 | -0.099 | -0.4781 | No |
| 232 | DNMT1 |  |  | 6455 | -0.099 | -0.4777 | No |
| 233 | UNG |  |  | 6479 | -0.100 | -0.4797 | No |
| 234 | CDC5L |  |  | 6486 | -0.100 | -0.4796 | No |
| 235 | NR1D2 |  |  | 6487 | -0.101 | -0.4788 | No |
| 236 | HSDL2 |  |  | 6505 | -0.101 | -0.4800 | No |
| 237 | SMC4 |  |  | 6517 | -0.102 | -0.4805 | No |
| 238 | EIF3M |  |  | 6532 | -0.104 | -0.4813 | No |
| 239 | MCM5 |  |  | 6553 | -0.105 | -0.4828 | No |
| 240 | DBF4 |  |  | 6572 | -0.106 | -0.4841 | No |
| 241 | MBIP |  |  | 6582 | -0.107 | -0.4843 | No |
| 242 | NUDT5 |  |  | 6590 | -0.107 | -0.4843 | No |
| 243 | OLIG2 |  |  | 6595 | -0.108 | -0.4839 | No |
| 244 | MAD2L1 |  |  | 6600 | -0.108 | -0.4835 | No |
| 245 | VRK1 |  |  | 6651 | -0.111 | -0.4885 | No |
| 246 | ANP32E |  |  | 6655 | -0.111 | -0.4880 | No |
| 247 | RPA2 |  |  | 6705 | -0.114 | -0.4928 | No |
| 248 | INPP5F |  |  | 6724 | -0.115 | -0.4940 | No |
| 249 | PHF10 |  |  | 6762 | -0.118 | -0.4974 | No |
| 250 | PRPS2 |  |  | 6830 | -0.123 | -0.5043 | No |
| 251 | LSM6 |  |  | 6880 | -0.128 | -0.5090 | No |
| 252 | APEX1 |  |  | 6898 | -0.129 | -0.5100 | No |
| 253 | ARL4A |  |  | 6930 | -0.131 | -0.5126 | No |
| 254 | ZEB1 |  |  | 6944 | -0.132 | -0.5131 | No |
| 255 | MMAB |  |  | 6963 | -0.134 | -0.5141 | No |
| 256 | CCDC59 |  |  | 7079 | -0.144 | -0.5265 | No |
| 257 | RBBP8 |  |  | 7099 | -0.145 | -0.5276 | No |
| 258 | PTPRN2 |  |  | 7125 | -0.148 | -0.5293 | No |
| 259 | SRRM1 |  |  | 7157 | -0.151 | -0.5318 | No |
| 260 | TMEM263 |  |  | 7170 | -0.153 | -0.5320 | No |
| 261 | FEN1 |  |  | 7201 | -0.155 | -0.5343 | No |
| 262 | GMNN |  |  | 7224 | -0.158 | -0.5356 | No |
| 263 | MRPL24 |  |  | 7245 | -0.161 | -0.5367 | No |
| 264 | EIF3E |  |  | 7253 | -0.162 | -0.5362 | No |
| 265 | DDAH1 |  |  | 7257 | -0.162 | -0.5353 | No |
| 266 | TMPO |  |  | 7296 | -0.167 | -0.5385 | No |
| 267 | ZFP36L2 |  |  | 7317 | -0.169 | -0.5395 | No |
| 268 | GPATCH4 |  |  | 7330 | -0.171 | -0.5395 | No |
| 269 | TOX3 |  |  | 7342 | -0.173 | -0.5395 | No |
| 270 | NAA50 |  |  | 7357 | -0.175 | -0.5397 | No |
| 271 | EIF2S1 |  |  | 7366 | -0.177 | -0.5393 | No |
| 272 | MCM6 |  |  | 7375 | -0.178 | -0.5388 | No |
| 273 | RDX |  |  | 7397 | -0.181 | -0.5399 | No |
| 274 | USP1 |  |  | 7398 | -0.181 | -0.5385 | No |
| 275 | CUTA |  |  | 7403 | -0.181 | -0.5375 | No |
| 276 | CEBPZ |  |  | 7404 | -0.181 | -0.5361 | No |
| 277 | CCT3 |  |  | 7464 | -0.187 | -0.5415 | No |
| 278 | ZFP36L1 |  |  | 7466 | -0.188 | -0.5402 | No |
| 279 | HNRNPA3 |  |  | 7470 | -0.188 | -0.5391 | No |
| 280 | GNL3 |  |  | 7548 | -0.197 | -0.5465 | No |
| 281 | MCM3 |  |  | 7552 | -0.198 | -0.5453 | No |
| 282 | ANXA7 |  |  | 7564 | -0.199 | -0.5451 | No |
| 283 | LYRM2 |  |  | 7591 | -0.203 | -0.5465 | No |
| 284 | GLUL |  |  | 7594 | -0.203 | -0.5452 | No |
| 285 | BIRC5 |  |  | 7596 | -0.204 | -0.5437 | No |
| 286 | SALL1 |  |  | 7597 | -0.204 | -0.5421 | No |
| 287 | GLTP |  |  | 7609 | -0.205 | -0.5418 | No |
| 288 | DERA |  |  | 7626 | -0.207 | -0.5420 | No |
| 289 | HAUS1 |  |  | 7761 | -0.227 | -0.5559 | No |
| 290 | AIF1L |  |  | 7762 | -0.227 | -0.5541 | No |
| 291 | FBL |  |  | 7780 | -0.230 | -0.5543 | No |
| 292 | CENPU |  |  | 7871 | -0.248 | -0.5629 | No |
| 293 | DHFR |  |  | 7878 | -0.250 | -0.5617 | No |
| 294 | SSB |  |  | 7906 | -0.257 | -0.5628 | No |
| 295 | ZFAND6 |  |  | 7909 | -0.257 | -0.5610 | No |
| 296 | TIMM10 |  |  | 7925 | -0.261 | -0.5608 | No |
| 297 | CKS2 |  |  | 7972 | -0.270 | -0.5640 | Yes |
| 298 | HMGB2 |  |  | 7973 | -0.271 | -0.5619 | Yes |
| 299 | HIST1H1C |  |  | 7978 | -0.271 | -0.5602 | Yes |
| 300 | BBX |  |  | 7987 | -0.272 | -0.5591 | Yes |
| 301 | PRDX3 |  |  | 8005 | -0.277 | -0.5589 | Yes |
| 302 | TMSB15A |  |  | 8046 | -0.284 | -0.5613 | Yes |
| 303 | RFC2 |  |  | 8049 | -0.285 | -0.5593 | Yes |
| 304 | CHCHD3 |  |  | 8055 | -0.287 | -0.5577 | Yes |
| 305 | PCNA |  |  | 8082 | -0.293 | -0.5584 | Yes |
| 306 | FAM114A1 |  |  | 8104 | -0.298 | -0.5585 | Yes |
| 307 | DPH3 |  |  | 8113 | -0.299 | -0.5571 | Yes |
| 308 | DUT |  |  | 8118 | -0.300 | -0.5553 | Yes |
| 309 | CCT5 |  |  | 8125 | -0.302 | -0.5536 | Yes |
| 310 | TMEM106C |  |  | 8146 | -0.308 | -0.5535 | Yes |
| 311 | CDK6 |  |  | 8156 | -0.310 | -0.5522 | Yes |
| 312 | CBX5 |  |  | 8171 | -0.318 | -0.5513 | Yes |
| 313 | PARP1 |  |  | 8182 | -0.321 | -0.5500 | Yes |
| 314 | H2AFY |  |  | 8189 | -0.323 | -0.5481 | Yes |
| 315 | SRSF3 |  |  | 8191 | -0.323 | -0.5457 | Yes |
| 316 | CKS1B |  |  | 8241 | -0.336 | -0.5488 | Yes |
| 317 | LDHA |  |  | 8263 | -0.343 | -0.5486 | Yes |
| 318 | HNRNPUL1 |  |  | 8269 | -0.345 | -0.5465 | Yes |
| 319 | COA4 |  |  | 8305 | -0.356 | -0.5478 | Yes |
| 320 | SNRPC |  |  | 8316 | -0.359 | -0.5462 | Yes |
| 321 | HNRNPF |  |  | 8319 | -0.360 | -0.5436 | Yes |
| 322 | PA2G4 |  |  | 8320 | -0.360 | -0.5408 | Yes |
| 323 | LITAF |  |  | 8324 | -0.361 | -0.5383 | Yes |
| 324 | SOX9 |  |  | 8327 | -0.361 | -0.5357 | Yes |
| 325 | GLO1 |  |  | 8338 | -0.364 | -0.5340 | Yes |
| 326 | CLIC1 |  |  | 8368 | -0.378 | -0.5344 | Yes |
| 327 | LDHB |  |  | 8372 | -0.379 | -0.5318 | Yes |
| 328 | WWTR1 |  |  | 8384 | -0.383 | -0.5301 | Yes |
| 329 | RSL1D1 |  |  | 8388 | -0.385 | -0.5275 | Yes |
| 330 | NAP1L1 |  |  | 8404 | -0.389 | -0.5262 | Yes |
| 331 | HNRNPC |  |  | 8413 | -0.392 | -0.5240 | Yes |
| 332 | HSPD1 |  |  | 8418 | -0.393 | -0.5214 | Yes |
| 333 | HNRNPD |  |  | 8436 | -0.402 | -0.5203 | Yes |
| 334 | LSM2 |  |  | 8445 | -0.405 | -0.5180 | Yes |
| 335 | CKB |  |  | 8448 | -0.407 | -0.5151 | Yes |
| 336 | EEF1D |  |  | 8455 | -0.412 | -0.5126 | Yes |
| 337 | RPS10 |  |  | 8475 | -0.421 | -0.5115 | Yes |
| 338 | NCL |  |  | 8496 | -0.433 | -0.5104 | Yes |
| 339 | NUCKS1 |  |  | 8502 | -0.435 | -0.5076 | Yes |
| 340 | PEA15 |  |  | 8509 | -0.439 | -0.5049 | Yes |
| 341 | RAD23A |  |  | 8524 | -0.446 | -0.5030 | Yes |
| 342 | ALYREF |  |  | 8535 | -0.449 | -0.5007 | Yes |
| 343 | SNRPD1 |  |  | 8537 | -0.451 | -0.4973 | Yes |
| 344 | RAN |  |  | 8555 | -0.463 | -0.4957 | Yes |
| 345 | CNBP |  |  | 8565 | -0.466 | -0.4931 | Yes |
| 346 | HNRNPA1 |  |  | 8570 | -0.469 | -0.4899 | Yes |
| 347 | NME1 |  |  | 8573 | -0.471 | -0.4864 | Yes |
| 348 | HSPB1 |  |  | 8586 | -0.478 | -0.4841 | Yes |
| 349 | RPL31 |  |  | 8592 | -0.482 | -0.4809 | Yes |
| 350 | PABPC1 |  |  | 8598 | -0.488 | -0.4776 | Yes |
| 351 | TYMS |  |  | 8599 | -0.488 | -0.4738 | Yes |
| 352 | YBX3 |  |  | 8604 | -0.493 | -0.4704 | Yes |
| 353 | HMGN2 |  |  | 8608 | -0.496 | -0.4669 | Yes |
| 354 | CDK4 |  |  | 8625 | -0.510 | -0.4648 | Yes |
| 355 | ANXA5 |  |  | 8626 | -0.510 | -0.4608 | Yes |
| 356 | HMGB1 |  |  | 8653 | -0.527 | -0.4597 | Yes |
| 357 | NUTF2 |  |  | 8677 | -0.544 | -0.4581 | Yes |
| 358 | SLC25A5 |  |  | 8681 | -0.546 | -0.4542 | Yes |
| 359 | SNRPB |  |  | 8683 | -0.547 | -0.4501 | Yes |
| 360 | NPM1 |  |  | 8706 | -0.566 | -0.4482 | Yes |
| 361 | SRI |  |  | 8709 | -0.572 | -0.4440 | Yes |
| 362 | RPL13A |  |  | 8714 | -0.578 | -0.4399 | Yes |
| 363 | SNRPD2 |  |  | 8718 | -0.581 | -0.4357 | Yes |
| 364 | JUND |  |  | 8723 | -0.586 | -0.4316 | Yes |
| 365 | ENO1 |  |  | 8734 | -0.593 | -0.4281 | Yes |
| 366 | PRDX2 |  |  | 8749 | -0.611 | -0.4250 | Yes |
| 367 | RPL10A |  |  | 8765 | -0.623 | -0.4219 | Yes |
| 368 | DEK |  |  | 8770 | -0.627 | -0.4174 | Yes |
| 369 | ANAPC11 |  |  | 8773 | -0.630 | -0.4127 | Yes |
| 370 | H2AFZ |  |  | 8774 | -0.630 | -0.4078 | Yes |
| 371 | RPL5 |  |  | 8780 | -0.635 | -0.4034 | Yes |
| 372 | RPL23 |  |  | 8781 | -0.636 | -0.3984 | Yes |
| 373 | RPL7 |  |  | 8792 | -0.644 | -0.3946 | Yes |
| 374 | GSTP1 |  |  | 8795 | -0.647 | -0.3897 | Yes |
| 375 | RPL21 |  |  | 8802 | -0.655 | -0.3853 | Yes |
| 376 | SET |  |  | 8813 | -0.664 | -0.3813 | Yes |
| 377 | MZT2B |  |  | 8816 | -0.665 | -0.3763 | Yes |
| 378 | RANBP1 |  |  | 8819 | -0.669 | -0.3713 | Yes |
| 379 | NME4 |  |  | 8824 | -0.678 | -0.3665 | Yes |
| 380 | RPL10 |  |  | 8835 | -0.690 | -0.3622 | Yes |
| 381 | RPL27A |  |  | 8838 | -0.696 | -0.3570 | Yes |
| 382 | RPL27 |  |  | 8840 | -0.698 | -0.3517 | Yes |
| 383 | RPL37A |  |  | 8860 | -0.721 | -0.3483 | Yes |
| 384 | RPL15 |  |  | 8863 | -0.724 | -0.3428 | Yes |
| 385 | RPS27 |  |  | 8865 | -0.727 | -0.3373 | Yes |
| 386 | GNG5 |  |  | 8866 | -0.728 | -0.3316 | Yes |
| 387 | RHOA |  |  | 8869 | -0.734 | -0.3261 | Yes |
| 388 | RPS27A |  |  | 8875 | -0.742 | -0.3208 | Yes |
| 389 | RPL35 |  |  | 8892 | -0.763 | -0.3167 | Yes |
| 390 | RPL39 |  |  | 8893 | -0.763 | -0.3108 | Yes |
| 391 | PTMA |  |  | 8894 | -0.764 | -0.3048 | Yes |
| 392 | CHCHD2 |  |  | 8898 | -0.770 | -0.2991 | Yes |
| 393 | RPLP0 |  |  | 8904 | -0.777 | -0.2936 | Yes |
| 394 | RPS28 |  |  | 8906 | -0.778 | -0.2876 | Yes |
| 395 | RPL18 |  |  | 8907 | -0.780 | -0.2815 | Yes |
| 396 | RPS25 |  |  | 8909 | -0.787 | -0.2755 | Yes |
| 397 | BTF3 |  |  | 8911 | -0.789 | -0.2694 | Yes |
| 398 | PPIA |  |  | 8913 | -0.797 | -0.2633 | Yes |
| 399 | GAPDH |  |  | 8915 | -0.803 | -0.2571 | Yes |
| 400 | RPL3 |  |  | 8916 | -0.803 | -0.2509 | Yes |
| 401 | RPS16 |  |  | 8917 | -0.812 | -0.2445 | Yes |
| 402 | RPL9 |  |  | 8918 | -0.813 | -0.2381 | Yes |
| 403 | SERF2 |  |  | 8920 | -0.816 | -0.2319 | Yes |
| 404 | RPS4X |  |  | 8923 | -0.826 | -0.2256 | Yes |
| 405 | RPS18 |  |  | 8926 | -0.829 | -0.2194 | Yes |
| 406 | RPS6 |  |  | 8929 | -0.837 | -0.2131 | Yes |
| 407 | RPL36 |  |  | 8931 | -0.843 | -0.2066 | Yes |
| 408 | RPLP2 |  |  | 8932 | -0.844 | -0.2000 | Yes |
| 409 | NACA |  |  | 8933 | -0.844 | -0.1934 | Yes |
| 410 | RPL23A |  |  | 8934 | -0.845 | -0.1868 | Yes |
| 411 | RPS3 |  |  | 8936 | -0.850 | -0.1802 | Yes |
| 412 | TPI1 |  |  | 8939 | -0.852 | -0.1738 | Yes |
| 413 | RPS19 |  |  | 8941 | -0.855 | -0.1672 | Yes |
| 414 | RPL6 |  |  | 8942 | -0.856 | -0.1605 | Yes |
| 415 | RPL13 |  |  | 8944 | -0.862 | -0.1539 | Yes |
| 416 | RPL12 |  |  | 8945 | -0.864 | -0.1472 | Yes |
| 417 | YBX1 |  |  | 8948 | -0.872 | -0.1406 | Yes |
| 418 | RPS14 |  |  | 8949 | -0.877 | -0.1337 | Yes |
| 419 | RPL4 |  |  | 8950 | -0.881 | -0.1268 | Yes |
| 420 | UQCRH |  |  | 8951 | -0.885 | -0.1199 | Yes |
| 421 | RPL32 |  |  | 8953 | -0.891 | -0.1130 | Yes |
| 422 | RPS13 |  |  | 8956 | -0.906 | -0.1062 | Yes |
| 423 | RPS3A |  |  | 8957 | -0.906 | -0.0991 | Yes |
| 424 | EEF1A1 |  |  | 8958 | -0.915 | -0.0919 | Yes |
| 425 | PKM |  |  | 8959 | -0.915 | -0.0848 | Yes |
| 426 | RPL7A |  |  | 8963 | -0.925 | -0.0779 | Yes |
| 427 | RPL29 |  |  | 8964 | -0.930 | -0.0706 | Yes |
| 428 | RPLP1 |  |  | 8968 | -0.958 | -0.0634 | Yes |
| 429 | RPL18A |  |  | 8969 | -0.964 | -0.0559 | Yes |
| 430 | RPS15 |  |  | 8970 | -0.965 | -0.0484 | Yes |
| 431 | RPL8 |  |  | 8971 | -0.992 | -0.0406 | Yes |
| 432 | RPSA |  |  | 8973 | -1.005 | -0.0328 | Yes |
| 433 | RPL41 |  |  | 8974 | -1.013 | -0.0249 | Yes |
| 434 | RPS8 |  |  | 8976 | -1.044 | -0.0169 | Yes |
| 435 | TUBA1B |  |  | 8980 | -1.103 | -0.0086 | Yes |
| 436 | TMSB4X |  |  | 8983 | -1.142 | 0.0001 | Yes |
Table: GSEA details [plain text format]

  

Fig 2: RICHARDS\_NOWAKOWSKI\_SCIENCE\_2017\_MGE\_RG2\_UPREG: Random ES distribution      
 Gene set null distribution of ES for **RICHARDS\_NOWAKOWSKI\_SCIENCE\_2017\_MGE\_RG2\_UPREG**
